# Transfemoral Compared to Transapical Transcatheter Aortic Valve Implantation in Chronic Dialysis-Dependent Patients

**DOI:** 10.3390/jcm14010135

**Published:** 2024-12-29

**Authors:** Norman Mangner, Manuela Schrader, Stephan Haussig, Philipp Kiefer, Sergey Leontyev, Utz Kappert, Konstantin Alexiou, Lisa Crusius, Sandra Erbs, Tomasz Gasior, Jean-Honoré Steul, Keita Goto, Anne Trausch, Jennifer Hommel, Mohamed Abdel-Wahab, Michael A. Borger, David Holzhey, Axel Linke, Felix J. Woitek

**Affiliations:** 1 Herzzentrum Dresden, Department of Internal Medicine and Cardiology, Technische Universität Dresden, Fetscherstr. 76, 01307 Dresden, Germanyfelix.woitek@tu-dresden.de (F.J.W.); 2 Department of Internal Medicine/Cardiology, Heart Center Leipzig, University of Leipzig, 04289 Leipzig, Germany; 3 Department of Cardiac Surgery, Heart Center Leipzig, University of Leipzig, 04289 Leipzig, Germany; 4 Herzzentrum Dresden, Department of Cardiac Surgery, Technische Universität Dresden, 01307 Dresden, Germany; 5 Faculty of Medicine, WSB University, 41-300 Dabrowa Gornicza, Poland

**Keywords:** aortic stenosis, dialysis, end-stage renal disease, TAVI, transfemoral, transapical

## Abstract

**Introduction:** Patients with end-stage kidney disease (ESRD) represent a high-risk population in terms of both development of and death by cardiovascular diseases. Outcome data of ESRD patients with severe aortic valve stenosis (AS) treated by transcatheter aortic valve implantation (AVI) are scarce. We aim to compare the outcome of ESRD patients undergoing transfemoral (TF) or transapical (TA) AVI. **Methods:** From June 2006 to December 2019, 176 consecutive patients with ESRD receiving chronic hemodialysis underwent TF- or TA-AVI at two German heart centers. The primary outcome measure was 1-year all-cause mortality. Other outcomes included VARC-3 defined device success and early safety. **Results:** The cohort comprised 61 (34.7%) patients receiving TA-AVI and 115 (65.3%) patients receiving TF-AVI. Perioperative risk, assessed using the EuroScore II, was not different between groups. VARC-3 defined device success (52.5% vs. 80.0%, *p* < 0.001) and early safety (27.9% vs. 45.2%, *p* = 0.025) were lower in TA-AVI patients compared to the TF-AVI group. The 30-day mortality was 4.7-fold higher in TA- compared TF-AVI patients (24.6% vs. 5.2%, *p* < 0.001). The 1-year mortality was higher in TA- compared with TF-AVI patients (57.3% vs. 27.8%, *p* < 0.001). By applying a Cox regression analysis, it was found that TA-AVI was the only independent factor associated with 1-year all-cause mortality (HR_adj_ 2.65 (95%-CI 1.63-4.30), *p* < 0.001). **Conclusions:** In ESRD patients, TA-AVI was associated with worse early outcomes and increased mortality up to 1 year compared to the TF-AVI. Transfemoral access is recommended, when feasible, in ESRD patients undergoing TAVI.

## 1. Introduction

Patients with end-stage kidney disease (ESRD) represent a high-risk population in terms of both development of and death from cardiovascular diseases [1]. Severe aortic stenosis (AS) is the most common valvular heart disease in this particular patient cohort [2]. However, surgical aortic valve replacement (SAVR) is associated with an increased risk of perioperative morbidity and mortality compared to patients without chronic kidney disease (CKD) or with less advanced CKD [2]. Transcatheter aortic valve implantation (TAVI) has become the standard of care in older patients (≥75 years of age) or in those who are at high risk (STS-PROM/EuroScore II > 8%) or unsuitable for SAVR [3]. However, ESRD patients have thus far been excluded from the major randomized controlled trials comparing TAVI vs. SAVR [4,5,6,7,8]. Observational data show that ESRD patients are associated with significantly higher rates of short- and long-term mortality, life-threatening and/or major bleeding, new permanent pacemaker implantation, and device failure compared with non-dialysis patients [9]. By comparing TAVI vs. SAVR, it has been found that ESRD patients have the same 1-year mortality rates, although survival after 30 days has been shown to be higher in patients treated with TAVI according to a German Aortic Valve Registry analysis [10]. In the TAVI cohort of this analysis, 30.1% of the patients received the intervention via a transapical access [10], which is associated with a higher risk of complications and mortality in the overall TAVI population [11]. Data comparing those two access sites among ESRD patients are scarce. Thus, we aim to compare the baseline and procedural characteristics and the outcome of ESRD patients undergoing transfemoral (TF) or transapical (TA) AVI.

## 2. Methods

### 2.1. Patient Cohort

From June 2006 to December 2019, 176 consecutive patients with ESRD receiving hemodialysis therapy were treated using a transfemoral or transapical approach at two German heart centers. All cases were discussed in the heart team and the choice of access was made on institutional preferences and clinical factors including imaging studies. Baseline characteristics and procedural and outcome data were prospectively collected. Follow-up was performed after 30 days and 12 months. Presence of lung disease was defined according to the EuroScore definition [12]. Immunosuppressant medication, diabetes mellitus, coronary artery disease, and peripheral artery disease were defined according to the STS database [13]. The registry was approved by the Ethics Committee at the University of Leipzig (registration number: 167-10-12072010, 12 July 2010) and by the Ethics Committee at the Technical University Dresden (EK 41012019, 15 January 2019).

### 2.2. Outcome Measures

The primary outcome measure was the 1-year all-cause mortality. Other outcome measures included technical success, device success, and early safety as defined by VARC-3 [14]. Technical success is a composite of (i) freedom from mortality, (ii) successful access, delivery of the device, and retrieval of the delivery system, (iii) correct positioning of a single prosthetic heart valve into the proper anatomical location, and (iv) freedom from surgery or intervention related to the device, freedom from a major vascular, access-related, or cardiac structural complication following exit from the operation room. Device success is a composite of technical success and intended performance of the valve (mean gradient < 20 mmHg, peak velocity < 3 m/s, Doppler velocity index 0.25, and less than moderate aortic regurgitation) after 30 days. Early safety is a composite of (i) freedom from all-cause mortality, (ii) freedom from all stroke, (iii) freedom from VARC type 2–4 bleeding, (iv) freedom from major vascular, access-related, or cardiac structural complication, (v) freedom from acute kidney injury stage 3 or 4, (vi) freedom from moderate or severe aortic regurgitation, (vii) freedom from new permanent pacemaker due to procedure-related conduction abnormalities, and (viii) freedom from surgery or intervention related to the device after 30 days. The single components 30-day mortality, myocardial infarction, stroke, bleeding, and access site complications were also assessed.

### 2.3. Statistical Analysis

The statistical analysis was performed using the SPSS Statistics software, version 27.0 (IBM Corporation, Armonk, NY, USA). Categorical variables are expressed as numbers and percentage and were compared through the use of the chi-squared test or Fisher’s exact test, as appropriate. Continuous variables are expressed as the median with the corresponding 25th and 75th percentile and were compared using the Mann–Whitney U test due to a non-normal distribution assessed using the Shapiro–Wilk test.

Predictors of the composite outcome measures, i.e., device success and early safety, were evaluated using a binary logistic regression analysis. Clinically relevant baseline variables with a *p*-value ≤ 0.1 in the context of univariate analysis were included after correcting for collinearity. Age and sex were forced into the models.

Thirty-day and 1-year mortality were analyzed according to the Kaplan–Meier method, and group comparisons were made by conducting the log-rank test. Independent predictors of 1-year all-cause mortality were determined using a Cox proportional hazards regression model. Clinically relevant variables with a *p*-value ≤ 0.1 in the context of univariate analysis were included in the model after correcting for collinearity. Collinearity was assumed if R was greater than 0.70 in the bivariate correlation test, the tolerance value was below 0.10, and/or the variable inflation factor (VIF) was greater than 10. Missing values were not included in the model. Age and sex were forced into the model. A two-sided *p*-value < 0.05 was considered to be significant.

## 3. Results

### 3.1. Baseline Characteristics and Procedural Data

The cohort comprised 61 (34.7%) patients receiving TA-AVI and 115 (65.3%) patients receiving TF-AVI. Baseline and procedural characteristics are shown in Table 1. ESRD patients receiving TA-AVI were more likely to have a history of prior coronary artery bypass grafting and were more likely associated with a higher incidence of peripheral artery disease, but a lower incidence rate of diabetes mellitus and a higher left ventricular ejection fraction (all *p* < 0.05). Perioperative risk, assessed using the EuroScore II, was not different between the groups. TA-AVI patients more often received a balloon-expandable, intra-annular prosthesis resulting in a higher mean gradient and a smaller aortic valve area. The rate of moderate/severe paravalvular aortic regurgitation was comparable between TA- and TF-AVI-treated ESRD patients. The procedure time was longer but the amount of contrast dye was lower in the TA-AVI group vs. the TF-AVI group.

### 3.2. Outcome Data After 30 Days

While technical success was comparable between groups, VARC-3 defined device success and early safety were lower in the TA-AVI group compared to the TF-AVI group (Table 2). The lower device success in the TA-AVI group was primarily driven by a significantly higher 30-day mortality (24.6% vs. 5.2%), while the reduced early safety was related to the significantly higher rates of life-threatening/major bleeding (44.1% vs. 25.7%) (Table 2). There were no significant differences with regard to myocardial infarction, stroke, and access site complications. The rate of new permanent pacemaker/implantable cardioverter defibrillator implantation was lower in the TA-AVI group compared to the TF-AVI group (18.9% vs. 38.1%).

Independent predictors for device success and early safety are shown in Table 3 and Table 4. Device success was associated with previous PCI (OR 0.41, 95%-CI 0.19; 0.89) and TA-AVI (OR 0.24, 95%-CI 0.12; 0.49). Early safety was linked to male sex (OR 0.47 (95%-CI 0.23; 0.98) and NYHA III/IV at presentation (OR 0.40, 95%-CI 0.17; 0.94), whereas TA-AVI was not significantly related to lower early safety (OR 0.57, 95%-CI 0.28; 1.16).

### 3.3. All-Cause Mortality After 1 Year and Its Predictors

**Figure 1:**
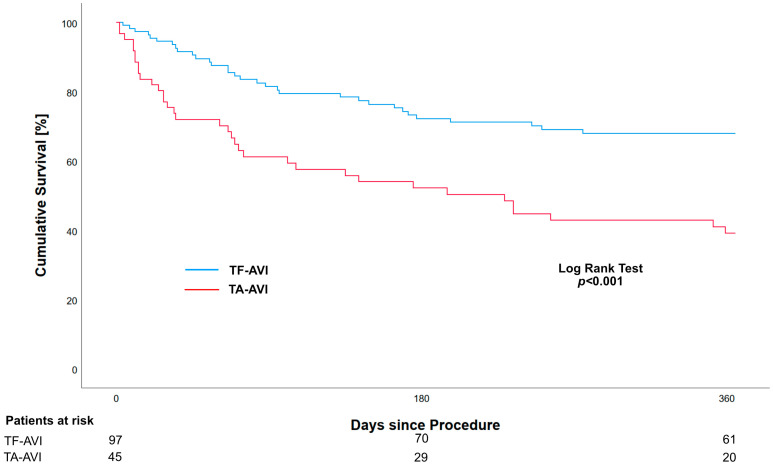
Kaplan–Meier analysis showing unadjusted all-cause 1-year survival in ESRD patients who have received transapical (TA-AVI) or transfemoral (TF-AVI) transcatheter aortic valve implantation.

**Figure 2:**
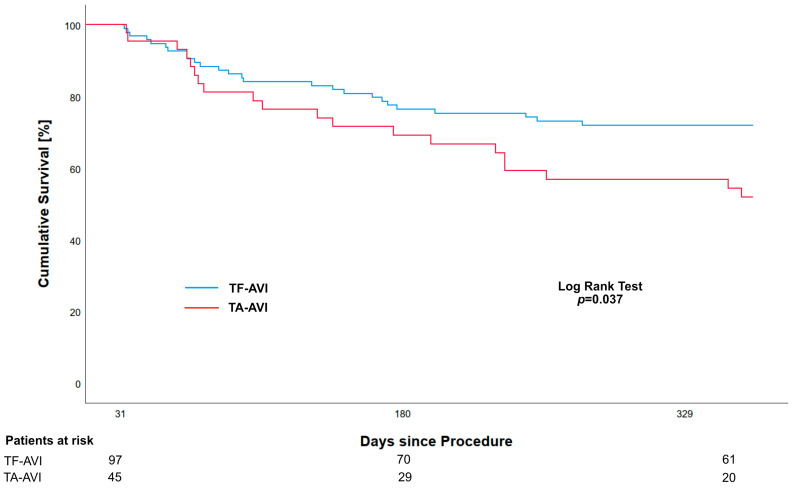
Landmark Kaplan–Meier analysis showing unadjusted all-cause 1-year survival starting on day 31 in ESRD patients who have received transapical (TA-AVI) or transfemoral (TF-AVI) transcatheter aortic valve implantation.

The cumulative mortality after 1 year was higher in the TA-AVI group compared to the TF-AVI group (57.3% vs. 27.8%, *p* < 0.001). The Kaplan–Meier estimates for the 1-year survival were 39.1% (95%-CI 28.2; 54.2) and 68.0% (95%-CI 59.4; 77.9) for the TA-AVI and TF-AVI groups, respectively (Figure 1). A landmark analysis starting on day 31 revealed an ongoing effect on mortality, with higher rates in the TA-AVI group compared to the TF-AVI group (44.4% vs. 26.8%, *p* = 0.037) (Figure 2).

By applying a Cox regression analysis, it was found that TA-AVI was the only independent factor associated with 1-year all-cause mortality (HR_adj_ 2.65 (95%-CI 1.63-4.30), *p* < 0.001) (Table 5).

## 4. Discussion

In this retrospective analysis, we found that ESRD patients undergoing TAVI by transapical access had lower VARC-3 defined device success and early safety rates compared to patients undergoing TAVI by transfemoral access. In addition, the 1-year all-cause mortality was 2.1-fold higher among ESRD patients undergoing TAVI by transapical access compared to ESRD patients receiving TAVI by transfemoral access.

ESRD patients are at high risk of both developing and experiencing complications during or after the treatment for cardiovascular diseases, in particular with regard to aortic stenosis [15,16]. Preexisting chronic kidney disease is common in patients undergoing TAVI; however, ESRD patients have been traditionally excluded from major trials comparing TAVI vs. SAVR [4,5,6,7,8]. Data from observational registries and a meta-analysis suggest that 1.8–4.3% of patients undergoing TAVI have ESRD [9,10,16,17,18]. With the increase in the risk factors for the development of ESRD in the population, in particular diabetes mellitus [19], one can expect an increase in those numbers in the future.

The best treatment option for aortic stenosis in ESRD patients is unknown. In general, short- and long-term mortality after TAVI [16] and SAVR [20] is much higher in ESRD patients compared to patients without kidney disease, indicating that the basic disease is an important modifier of the TAVI treatment effect. A TVT registry analysis determined the outcome of patients with ESRD receiving TAVI. Compared to the non-dialysis patients, ESRD patients were younger (76 years vs. 83 years; *p* < 0.01) and exhibited higher rates of comorbidities leading to a higher STS-predicted risk of mortality (median 13.5% vs. 6.2%; *p* < 0.01). Moreover, ESRD patients were associated with a higher in-hospital mortality (5.1% vs. 3.4%; *p* < 0.01), a higher rate of major bleeding (1.4% vs. 1.0%; *p* = 0.03), and a similar rate of major vascular complications and strokes. The 1-year mortality was significantly higher among the dialysis patients (36.8% vs. 18.7%; *p* < 0.01). According to the authors, the high 1-year survival raises concerns regarding the diminished benefit in this population [16]. While short-term mortality after TAVI improved over time, the long-term mortality remained disturbingly high among ESRD patients [21].

By comparing TAVI to SAVR, the observational data from the GARY registry show that mortality after 1 year was the same among TAVI (33.4%) and SAVR patients (35.0%, *p* = 0.72, IPTW-adjusted), while it was lower among patients who had undergone TAVI after 30 days in the ESRD patients (8.6% vs. 15.0%, *p* = 0.02, IPTW-adjusted), suggesting that TAVI may improve periprocedural outcomes [10]. In another analysis of ESRD patients from the U.S. using the Nationwide Inpatient Sample database, TAVI was associated with a lower hospital mortality rate, less frequent blood cell transfusions, lower resource utilization, and lower costs compared to SAVR [22].

Against the background of this high mortality among ESRD patients undergoing TAVI, it is of the utmost importance to perform the procedure in its safest and most effective way. Regarding the access route for TAVI, two sub-analyses of the aforementioned studies indicate that TA, or alternative access, is associated with higher short- and midterm mortality rates among ESRD patients [10,16]. Therefore, we analyzed ESRD patients undergoing TAVI with a transfemoral versus transapical access in two high performing German TAVI centers. Baseline characteristics including age, sex, perioperative risk, and comorbidities compare well to those from other cohorts of ESRD patients [10,16]. We found a lower efficacy and safety of the TAVI procedure when performed transapically, mainly due to a higher rate of life-threatening/major bleeding, potentially contributing to the 4.7-fold higher 30-day mortality in the TA-AVI group. The stroke rate was also more than doubled in the TA-AVI group, although it failed to reach statistical significance due to the low absolute numbers. Besides the procedure itself, atrial fibrillation, known to be associated with the occurrence of left atrial appendage thrombosis in TAVI patients [23], is a common comorbidity in ESRD patients and was evident in 43.8% of the patients at baseline in our cohort. In general, the optimal anticoagulatory therapy used in ESRD patients with atrial fibrillation is still a matter of debate [24], with no data on ESRD patients who have been treated with TAVI. The rate of new permanent pacemaker/implantable cardioverter defibrillator implantation was lower in the TA-AVI group compared to the TF-AVI group, a phenomenon which is most likely related to a significantly higher use of self-expanding TAVI prostheses among TF-AVI patients compared to TA-AVI patients [25].

According to our analysis, the choice of access not only affects early outcomes, but also 1-year mortality rates indicated by the landmark analysis, with TA-AVI being the only independent predictor of 1-year mortality. Therefore, there appears to be a persistent effect of the initial operative trauma on mortality. Our data and those of others highlight the fact that the class I recommendation to use a transfemoral TAVI access also extends to ESRD patients [3]. In the general TAVI population, the rate of TA-AVI has substantially decreased over the years, with the practice nearly no longer in use in the US (0.3% in 2019) [26]. However, an analysis using the German GARY registry has demonstrated that 30.1% of dialysis-dependent patients receive the TAVI procedure transapically, despite only 50% of them having a diagnosis of peripheral artery disease [10], suggesting that the transapical access is not used as an alternative access only. Moreover, these data suggest rethinking the first choice of an alternative access, with the transaxillary access showing promising results [27].

### Limitations

Our analysis has several limitations. Despite the fact that the data were analyzed from a prospective registry including real-world, consecutive patients, all bias inherent to a retrospectively evaluated, unmonitored registry has to be considered while interpreting these data. The choice of access was made by the treating physician and the specific reason for this decision was not documented in the database; therefore, it is possible that TA-AVI patients would have been ineligible for TF-AVI. However, peripheral artery disease did not appear as a predictor of any outcome. In general, in the early years, access choice was commonly related to separate programs in cardiology and cardiac surgery, whereas, in the later years, technical aspects and comorbidities determined the choice of access. The treatment period was from 2006 to 2019, a time in which TAVI experienced many technical and procedural changes. The effect of successive device iterations, improved imaging and patient selection, as well as operator experience is of importance when interpreting these results. Finally, the use of TA-AVI has decreased significantly over the last decade, and other alternative access options, e.g., axillary, have emerged. However, cases of ESRD patients treated using an alternative access other than TA are still limited, and, in particular, an axillary approach potentially endangers downstream hemodialysis shunts.

## 5. Conclusions

TA-AVI in ESRD patients is associated with worse early outcomes and increased mortality up to 1 year after the procedure compared to patients receiving TF-AVI. Whenever feasible, TF-AVI should be the primary approach in ESRD patients.

## Figures and Tables

**Table 1:** Baseline and procedural characteristics.

	All Patients *n* = 176	TF-AVI *n* = 115	TA-AVI *n* = 61	*p*-Value
Age (years)	78 (72; 81)	78 (72; 81)	78 (72; 81)	0.547
Male gender, *n* (%)	109/176 (61.9)	68 (59.1)	41 (67.2)	0.293
Body mass index (kg/m²)	26.6 (23.6; 30.6)	27.2 (24.0; 31.4)	25.7 (22.7; 28.4)	0.010
EuroScore II (%)	7.0 (4.4; 11.7) *n* = 173	7.2 (4.6; 11.6) *n* = 112	6.6 (4.2; 12.1) *n* = 61	0.700
New York Heart Association class III/IV, *n* (%)	145/173 (83.8)	95/112 (84.8)	50/61 (82.0)	0.626
Coronary artery disease, *n* (%)	108/176 (61.4)	70/115 (60.9)	38/61 (62.3)	0.853
Previous percutaneous coronary intervention, *n* (%)	42/176 (23.9)	31/115 (27.0)	11/61 (18.0)	0.186
Previous coronary artery bypass surgery, *n* (%)	34/176 (19.3)	16/115 (13.9)	18/61 (29.5)	0.013
Atrial fibrillation/flutter, *n* (%)	77/176 (43.8)	51/115 (44.3)	26/61 (42.6)	0.826
Arterial hypertension, *n* (%)	168/176 (95.5)	109/115 (94.8)	59/61 (96.7)	0.716
Diabetes mellitus, *n* (%)	97/176 (55.1)	73/115 (63.5)	24/61 (39.3)	0.002
Previous stroke, *n* (%)	17/175 (9.7)	10/114 (8.8)	7/61 (11.5)	0.565
Peripheral artery disease, *n* (%)	46/176 (26.1)	24/115 (20.9)	22/61 (36.1)	0.029
Carotid disease, *n* (%)	42/172 (24.4)	27/111 (24.3)	15/61 (24.6)	0.969
Chronic obstructive lung disease, *n* (%)	38/176 (21.6)	20/115 (17.4)	18/61 (29.5)	0.063
Immunosuppressive therapy, *n* (%)	11/176 (6.3)	10/115 (8.7)	1/61 (1.6)	0.100
Left ventricular ejection fraction (%)	55 (40; 61) *n* = 166	52 (38; 60) *n* = 107	56 (44; 63) *n* = 59	0.042
Aortic valve area (cm²)	0.7 (0.6; 0.8) *n* = 164	0.7 (0.6; 0.8) *n* = 105	0.7 (0.6; 0.8) *n* = 59	0.661
Mean gradient (mmHg)	40 (28; 51) *n* = 163	39 (28; 50) *n* = 105	41 (30; 53) *n* = 58	0.557
Mitral regurgitation 2/3, *n* (%)	62/168 (36.9)	37/109 (33.9)	25/59 (42.4)	0.280
Type of valve				< 0.001
Self-expanding, *n* (%)	97/176 (55.1)	82/115 (71.3)	15/61 (24.6)	
Balloon-expandable, *n* (%)	79/176 (44.9)	33/115 (28.7)	46/61 (75.4)	
Indication				0.066
Native valve, *n* (%)	167/176 (94.9)	112/115 (97.4)	55/61 (90.2)	
Valve-in-valve, *n* (%)	9/176 (5.1)	3/115 (2.6)	6/61 (9.8)	
Procedure Time (min)	59 (43; 80) *n* = 167	52 (40; 69) *n* = 106	65 (51; 94) *n* = 61	< 0.001
Contrast dye (ml)	100 (75; 125) *n* = 131	115 (90; 135) *n* = 90	70 (60; 90) *n* = 41	< 0.001
Residual mean gradient (mmHg)	9 (7; 13) *n* = 153	8 (6; 12) *n* = 105	12 (7; 17) *n* = 48	0.002
Aortic valve area (cm²)	1.8 (1.5; 2.3) *n* = 93	1.9 (1.6; 2.4) *n* = 69	1.6 (1.3; 1.9) *n* = 24	0.007
Residual aortic regurgitation ≥ 2	10/156 (6.4)	6/107 (5.6)	4/49 (8.2)	0.507

Variables are expressed as numbers and percentages or median with their 25th and 75th quartiles.

**Table 2:** Procedural outcome.

	All Patients *n* = 176	TF-AVI *n* = 115	TA-AVI *n* = 61	*p*-Value
Technical success (VARC-3), *n* (%)	156/176 (88.6)	104/115 (90.4)	52/61 (85.2)	0.302
Device success (VARC-3), *n* (%)	124/126 (70.5)	92/115 (80.0)	32/61 (52.5)	< 0.001
Early safety (VARC-3), *n* (%)	69/176 (39.2)	52/115 (45.2)	17/61 (27.9)	0.025
Thirty-day mortality, *n* (%)	22/176 (12.5)	6/115 (5.2)	15/61 (24.6)	< 0.001
VARC myocardial infarction, *n* (%)	1/174 (0.6)	1/113 (0.9)	0/61 (0)	1.000
VARC stroke, *n* (%)	5/174 (2.9)	2/113 (1.8)	3/61 (4.9)	0.345
Major, *n* (%)	4/174 (2.3)	1/113 (0.9)	3/61 (4.9)	0.124
Minor, *n* (%)	1/174 (0.6)	1/113 (0.9)	0/61 (0)	1.000
VARC bleeding, *n* (%)	92/174 (52.9)	50/113 (44.2)	42/61 (68.9)	0.002
Life-threatening, *n* (%)	17/174 (9.8)	8/113 (7.1)	9/61 (4.8)	0.104
Major, *n* (%)	45/174 (25.9)	21/113 (18.6)	24/61 (39.3)	0.003
Minor, *n* (%)	30/174 (17.2)	21/113 (18.6)	9/61 (14.8)	0.523
VARC access site complication, *n* (%)	31/174 (17.8)	24/113 (21.2)	7/61 (11.5)	0.108
Major, *n* (%)	15/174 (8.6)	11/113 (9.7)	4/61 (6.6)	0.476
Minor, *n* (%)	11/174 (6.3)	10/113 (8.8)	1/62 (1.6)	0.099
Closure device failure, *n* (%)	8/174 (4.6)	3/113 (2.7)	2/61 (3.3)	1.000
New permanent pacemaker/implantable cardioverter *, *n* (%)	47/150 (31.3)	37/97 (38.1)	10/53 (18.9)	0.015

* excluding patients with pre-existing pacemaker/implantable cardioverters.

**Table 3:** Binary logistic regression. Univariate and multivariate predictors of device success.

	Univariate		Multivariate	
	OR (95%-CI)	*p*-Value	OR (95%-CI)	*p*-Value
Age (per 1 year increase)	1.03 (0.99; 1.08)	0.193	1.03 (0.98; 1.08)	0.256
Male sex	0.64 (0.32; 1.27)	0.198	1.16 (0.54; 2.45)	0.708
Body mass index (per 1 kg/m² increase)	1.05 (0.99; 1.05)	0.077	1.03 (0.97; 1.10)	0.312
EuroScore II (per 1% increase)	1.00 (0.96; 1.05)	0.855		
NYHA III/IV	0.92 (0.38; 2.24)	0.851		
Coronary artery disease	0.88 (0.45; 1.72)	0.711		
Previous myocardial infarction	0.88 (0.28; 2.78)	0.828		
Previous percutaneous coronary intervention	0.52 (0.25; 1.08)	0.078	0.41 (0.19; 0.89)	0.024
Previous coronary artery bypass surgery	1.46 (0.61; 3.48)	0.394		
Atrial fibrillation	1.72 (0.88; 3.36)	0.115		
Diabetes mellitus	1.50 (0.78; 2.87)	0.225		
Previous stroke	1.38 (0.43; 4.44)	0.593		
Peripheral artery disease	0.82 (0.40; 1.70)	0.597		
Chronic obstructive lung disease	1.23 (0.55; 2.75)	0.623		
Left ventricular ejection fraction (per 10% decrease)	1.16 (0.91; 1.48)	0.218		
Transapical vs. transfemoral	0.28 (0.14; 0.54)	< 0.001	0.24 (0.12; 0.49)	< 0.001
Balloon-expandable vs. self-expanding prosthesis	0.48 (0.25; 0.93)	0.028	0.92 (0.42; 2.04)	0.837

**Table 4:** Binary logistic regression. Univariate and multivariate predictors of early safety.

	Univariate		Multivariate	
	OR (95%-CI)	*p*-Value	OR (95%-CI)	*p*-Value
Age (per 1 year increase)	0.99 (0.95; 1.03)	0.616	0.97 (0.93; 1.02)	0.296
Male sex	0.62 (0.33; 1.16)	0.133	0.47 (0.23; 0.98)	0.043
Body mass index (per 1 kg/m² increase)	1.03 (0.98; 1.09)	0.204		
EuroScore II (per 1% increase)	1.01 (0.97; 1.05)	0.583		
NYHA III/IV	0.50 (0.22; 1.13)	0.095	0.40 (0.17; 0.94)	0.037
Coronary artery disease	0.97 (0.52; 1.80)	0.914		
Previous myocardial infarction	1.11 (0.37; 3.38)	0.853		
Previous percutaneous coronary intervention	0.72 (0.35; 1.49)	0.373		
Previous coronary artery bypass surgery	1.49 (0.70; 3.17)	0.298		
Atrial fibrillation	1.45 (0.79; 2.66)	0.236		
Diabetes mellitus	1.10 (0.60; 2.02)	0.763		
Previous stroke	0.82 (0.29; 2.34)	0.714		
Peripheral artery disease	0.78 (0.39; 1.56)	0.475		
Chronic obstructive lung disease	0.88 (0.42; 1.85)	0.736		
Left ventricular ejection fraction (per 10% decrease)	1.21 (0.97; 1.50)	0.095	1.26 (1.00; 1.59)	0.055
Transapical vs. transfemoral	0.47 (0.24; 0.91)	0.026	0.57 (0.28; 1.16)	0.121
Balloon-expandable vs. self-expanding prosthesis	1.34 (0.73; 2.46)	0.348		

**Table 5:** Cox regression analysis. Univariate and multivariate predictors of 1-year mortality.

	Univariate		Multivariate	
	HR (95%-CI)	*p*-Value	HR (95%-CI)	*p*-Value
Age (per 1 year increase)	1.02 (0.99; 1.06)	0.213	1.03 (1.00; 1.07)	0.077
Male sex	1.47 (0.87; 2.48)	0.150	1.24 (0.72; 2.16)	0.440
Body mass index (per 1 kg/m² increase)	0.98 (0.95; 1.02)	0.984		
EuroScore II (per 1% increase)	1.01 (0.98; 1.04)	0.629		
NYHA III/IV	1.31 (0.65; 2.65)	0.450		
Coronary artery disease	0.97 (0.59; 1.57)	0.886		
Previous myocardial infarction	1.12 (0.51; 2.47)	0.771		
Previous percutaneous coronary intervention	0.97 (0.55; 1.70)	0.906		
Previous coronary artery bypass surgery	1.74 (1.01; 2.98)	0.046	1.42 (0.81; 2.50)	0.219
Atrial fibrillation	0.90 (0.55; 1.45)	0.653		
Diabetes mellitus	0.52 (0.32; 0.84)	0.008	0.66 (0.40; 1.11)	0.121
Previous stroke	0.84 (0.36; 1.94)	0.682		
Peripheral artery disease	0.86 (0.49; 1.50)	0.591		
Chronic obstructive lung disease	1.32 (0.77; 2.26)	0.317		
Left ventricular ejection fraction (per 10% decrease)	1.09 (0.92; 1.29)	0.344		
Transapical vs. transfemoral	2.46 (1.52; 3.98)	<0.001	2.65 (1.63; 4.30)	<0.001
Balloon-expandable vs. self-expanding prosthesis	1.74 (1.07; 2.81)	0.026	1.23 (0.69; 2.17)	0.485

## Data Availability

The data underlying this paper will be shared upon reasonable request to the corresponding author and authors of each participating center.
